# Influence of Sudden Changes in Foot Strikes on Loading Rate Variability in Runners

**DOI:** 10.3390/s24248163

**Published:** 2024-12-21

**Authors:** Maxime Chabot, Alexandre Thibault-Piedboeuf, Marie-Lyne Nault, Jean-Sébastien Roy, Philippe C. Dixon, Martin Simoneau

**Affiliations:** 1 Faculty of Medicine, Department of Kinesiology, Université Laval, Quebec City, QC G1V OA6, Canada; maxime.chabot.8@ulaval.ca (M.C.);; 2 Center for Interdisciplinary Research in Rehabilitation and Social Integration (Cirris), Quebec City, QC G1M 2S8, Canada; 3 Azrieli Research Center of the CHU Sainte-Justine, Montreal, QC H3T 1C5, Canada; 4 Faculty of Medicine, Department of Surgery, Université de Montréal, Montréal, QC H3T 1J4, Canada; 5 Faculty of Medicine, School of Rehabilitation Sciences, Université Laval, Quebec City, QC G1V 0A6, Canada; 6 Department of Kinesiology and Physical Education, McGill University, Montreal, QC H3A 0G4, Canada; phil.dixon@mcgill.ca

**Keywords:** impact loading, ground reaction forces, running biomechanics, training intervention

## Abstract

Foot strike patterns influence vertical loading rates during running. Running retraining interventions often include switching to a new foot strike pattern. Sudden changes in the foot strike pattern may be uncomfortable and may lead to higher step-to-step variability. This study evaluated the effects of running with an imposed and usual foot strike on vertical loading rate variability and amplitude. Twenty-seven participants (16 men and 11 women; age range: 18–30 years) ran on an instrumented treadmill with their usual foot strike for 10 min. Then, the participants were instructed to run with an unusual foot strike for 6 min. We calculated the vertical instantaneous and vertical average loading rates and their variances over 200 steps to quantify vertical loading rate variability. We also calculated the amplitude and variability of the shank acceleration peak using an inertial measurement unit. The vertical loading rate and shank acceleration peak amplitudes were higher when running with a rearfoot strike, regardless of the foot strike conditions (i.e., usual or imposed). The vertical loading rate and shank acceleration peak variability were higher when running with an imposed rearfoot strike than when running with a usual forefoot strike. No differences were found in the vertical loading rate and shank acceleration peak variabilities between the imposed forefoot strike and usual rearfoot strike conditions. This study offers compelling evidence that adopting an imposed (i.e., unusual) rearfoot strike amplifies loading rate and shank acceleration peak variabilities.

## 1. Introduction

Running is one of the most popular activities because it is easily accessible and offers many health benefits. Running improves cardiovascular health and metabolic function, reduces adiposity, and boosts aerobic fitness [1,2]. However, due to the repetitive impact, injuries occur frequently. The incidence of lower-extremity injuries during running ranges from 20% to 79% [3,4], with the most common injuries arising at the knee and below [3,5]. Impact loading variables, such as vertical ground reaction forces (GRFs) or shank acceleration peak measured with wearable sensors, have been associated with running injuries [6,7,8,9]. Vertical loading rates describe the increase in the vertical GRF during the stance phase. The vertical loading rate refers to the vertical average loading rate (VALR) or the vertical instantaneous loading rate (VILR). The VALR and the VILR represent the average and the maximal slope, respectively, of the most linear portion of the increase in vertical GRF during the early stance phase [9,10,11]. However, the relationship between impact loading metrics, such as the VALR, the VILR, and shank acceleration peak, and running injuries remains unclear [8,12,13]. Nonetheless, researchers have developed running pattern retraining to reduce impact loading as foot strike patterns influence the vertical loading rates [14,15]. For rearfoot strike, heel landing produces an immediate and distinct impact peak in the vertical GRF early in the stance phase, unlike forefoot and midfoot strike, where landing on the toe or midfoot does not produce a distinct impact peak in the early stance phase [16,17,18,19]. The local impact peak during rearfoot strike is associated with higher loading rates and running injuries [6,18,20,21], potentially contributing to a 2.5 times higher injury rate in this running type than forefoot runners [16].

Most interventions focusing on running pattern retraining aim to reduce impact forces to alleviate pain and reduce the risk of injuries during running [22,23,24]. Interventions may involve changing strike frequency [25], stride length [26], foot strike pattern [27,28], or adopting “softer” running [10,29] using visual feedback of the impact (i.e., vertical ground reaction force or tibial acceleration). Foot strike pattern interventions often consist of switching from a rearfoot strike to a forefoot strike to attenuate the vertical loading rate [17]. Indeed, a study reported three case series where VALR and VILR decreased between 11 and 35% after switching to a forefoot strike [27]. However, sudden changes in foot strike patterns lead to biomechanical differences that increase the load on specific musculoskeletal structures, which can be uncomfortable and may not result in immediate or lasting changes for longer runs [30]. Results from past studies suggest that running with a rearfoot strike (RFS) increases knee joint loading, while running with a forefoot strike (FFS) increases ankle joint loading [15]. Ankle soreness was reported during running pattern retraining intervention and after a one-month follow-up for habitual RFS runners with patellofemoral pain who transitioned to an FFS [24]. Thus, running pattern retraining can lead to discomfort.

Variations in motor performance across multiple repetitions of a specific movement or sports task define movement variability [31]. Studies suggest that performing motor skills with optimal variability leads to a healthy and adaptable state [32]. Below or beyond that optimal state, movement variability could cause injury. Low movement variability reflects repetitive loading on the same tissue, possibly leading to overuse injuries [31]. In contrast, high movement variability refers to increased exposure to erratic and unstable positions of unfamiliar movements and may lead to acute injuries [32]. Very few studies have evaluated the variability of the vertical loading rate during running [33,34]. There is inconclusive evidence that higher impact loading amplitude is associated with running injuries [13]. Indeed, some prospective and retrospective studies have found a positive relationship between loading rate amplitude and running injuries [6,21,35,36]. However, other prospective or retrospective studies have found no association between impact loading and running injuries [37,38,39]. Because running-related injuries arise from various individual characteristics such as biomechanics, muscle strength, flexibility, and environmental factors like training surfaces, footwear, and running volume, a multifactorial approach is essential to understanding the underlying causes of these injuries [39]. Quantifying loading rate variability could offer additional insights into impact loading during running, supplementing the information provided by loading rate amplitude. We suggest that quantifying the variability in vertical loading rate between impacts would reveal the runner’s ability to mitigate impact patterns and loading rates over time. No studies have assessed whether imposing a specific running strike pattern alters the vertical loading rate variability or shank acceleration peak variability. Low variability in loading rate or shank acceleration peak across foot strikes indicates consistent impact loading with each step, which can be detrimental as the repeated stress is concentrated on the same structure, increasing the risk of injury. Conversely, high variability in loading rate or shank acceleration peak can also be harmful, as it indicates that specific structures must absorb excessive stress. Meanwhile, the neuromuscular system may not be adequately prepared to handle the impact, also increasing the risk of injury. Hence, switching to a new foot strike pattern stresses the neuromuscular system [40], and movement variability increases when adopting a new sensorimotor pattern, which can accelerate muscle fatigue [31]. Therefore, a metric quantifying loading rate and shank acceleration peak variabilities should also be considered when assessing the factors causing running-related injuries. Some studies have compared the effects of running with a usual and imposed unusual foot strike [17,41,42,43]. Still, none have studied the variability of the loading rate or shank acceleration peak across foot strikes immediately after switching to an imposed unusual foot strike. In the current study, we calculated the vertical loading rate and shank acceleration peak to assess foot strike variability while running on the treadmill. Therefore, this study aimed to (1) quantify the amplitude and variability of the loading rate and shank acceleration peak across foot strikes during running and (2) evaluate the effects of running with an imposed unusual forefoot strike or an imposed unusual rearfoot strike on the loading rate and shank acceleration peak amplitudes and variabilities across several steps. We hypothesized that the vertical loading rate and shank acceleration peak variabilities across foot strikes would be higher when running with an unusual foot strike than when running with a usual foot strike, regardless of the foot strike pattern. We also hypothesized that running with a rearfoot strike (i.e., usual or imposed) would increase the amplitude of the loading rate and shank acceleration peak compared to running with a forefoot strike.

## 2. Materials and Methods

### 2.1. Participants

Twenty-seven recreational runners (16 male and 11 female) aged between 18 and 30 years old who ran at least 15 km per week, were free of current lower-limb injuries three months before data collection, and had no pain during data collection were recruited. The participants were recruited through advertisements at the university’s sports center. Some participants (n = 12) were track and field athletes trained to use forefoot strikes to optimize their performance. Participants provided written informed consent before participating. The Institutional Review Board (*CIUSSS de la Capitale-Nationale*, MP-13-2022-2335) reviewed and approved the study. Before data collection, participant characteristics, including biological sex, sociodemographic data, symptomatology, and running habits, were collected (see Table 1).

### 2.2. Experimental Protocol

Before data collection, we measured each participant’s body mass and height. During testing, participants ran at their self-selected pace for each condition, determined during the warm-up. Participants started with a 4 min warm-up containing 2 min of walking and then 2 min of running; this helped the participants find their self-selected running pace. During the warm-up, we slowly increased the treadmill speed, and the participants verbally indicated whether the speed matched their normal training speed. During data acquisition, participants performed 2 foot strike conditions: running with usual and unusual (imposed) foot strikes. First, participants ran for 10 min with their usual foot strike; we asked them to run as they naturally do during regular training sessions. Then, participants performed 1 trial while running with an imposed foot strike for 6 min. For the imposed foot strike condition, we asked the participants running with a usual rearfoot strike to land on their toes as the 1st contact with the ground to produce a forefoot strike. Conversely, for the imposed condition, we asked the participants running with a usual forefoot strike (or midfoot strike) to land with their heel as the 1st contact with the ground (i.e., rearfoot strike). The participants conducted a 2 min familiarization trial to practice running with the imposed foot strike. During the experiment, a 5 min rest was allowed after each trial to attenuate fatigue.

To identify participants’ usual foot strikes, we recorded foot strikes during the usual foot strike trial with an iPad-mounted camera (iPad mini, Apple, Cupertino, CA, USA, 120 Hz). The iPad was located ~2 m from the participants and ~0.5 m above the ground to record the foot landing through a sagittal view. We also recorded foot strikes during the imposed foot strike condition to ensure the participants adopted the imposed foot strike pattern. The usual foot strike pattern was determined by analyzing the video recordings. We analyzed a minimum of 10 steps between the third and fourth minutes of the trial. Strikes with the heel as the 1st contact with the ground were considered rearfoot strikes. Strikes with the toe as 1st contact with the ground were considered forefoot strikes. We classified participants landing simultaneously with the toe and heel as midfoot strikers and included these participants in the forefoot strike group. Overall, 20 participants ran with a usual forefoot strike, while 7 participants used a usual rearfoot strike (Table 1).

### 2.3. Data Analysis

During testing, participants ran with their common training shoes on an instrumented treadmill (Bertec Corp., Columbus, OH, USA), which acquired ground reaction forces data at a sampling rate of 1000 Hz. Twenty-four out of the twenty-seven participants also wore two IMUs (Xsens MTw Awinda, Xsens North America Inc., Los Angeles, CA, USA). The IMUs were attached to both shanks using elastic bands and adhesive tape. We located the IMUs on the shanks above the lateral malleolus and aligned them with the fibula because a previous study reported a better correlation with vertical load rates [44]. We recorded the IMU data using a custom-made MATLAB script (version 2020b, The Mathworks Inc., Natick, MA, USA). Before each trial, we calibrated the IMUs to align the reference frames of the IMU’s vertical axis with each shank’s longitudinal axis [8]. The participants raised their left and right legs sequentially for the calibration. We collected the IMU data at a sampling rate of 100 Hz. We resampled the IMU data at 1000 Hz to match the sampling rate of the ground reaction forces. Participants also performed a vertical jump on the instrumented treadmill at the beginning of each trial to synchronize the IMU data with GRF data recorded while on the treadmill. Then, to align the shank acceleration and GRF signals, we identified the peak values corresponding to the vertical jump in each time series and shifted the shank acceleration time series to remove the delay. We acquired the GRF using the VICON Nexus software (version 2.12.1, VICON motion systems, Oxford, UK). We analyzed the data using custom-made MATLAB scripts (version 2020b, The Mathworks Inc., Natick, MA, USA). We calculated the amplitude spectral density of the vertical GRF data to determine the cut-off frequency of the low-pass filter (Supplementary Materials). We filtered the GRF data using a zero-lag fourth-order low-pass Butterworth filter with a cut-off frequency of 20 Hz. We chose a vertical force threshold of 100 N to identify foot strike and toe-off [45,46].

**Figure 1:**
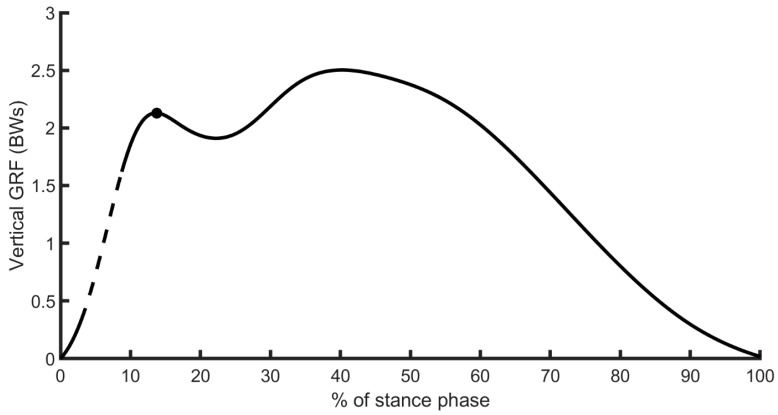
Typical vertical ground reaction force (GRF) for a rearfoot strike impact from one participant. The black dot represents the normalized vertical GRF peak. The dashed section describes the 20 to 80% region of the vertical impact peak used to calculate the vertical average loading rate (VALR) and vertical instantaneous loading rate (VILR).

To assess the effect of imposing a foot strike on participants, we computed the VALR and VILR from the GRF; both measures were normalized to body weight. A vertical impact peak was identified as a local maximum early in the stance phase, which is mainly observed during a rearfoot strike impact (Figure 1). We computed the VALR and VILR as the average and maximal slope between 20 and 80% of the vertical force from that vertical impact peak at each step [10]. In cases where the local maximum was missing from the vertical GRFs, typically during midfoot or forefoot strike, the force value at 15% of the stance phase was identified as the vertical impact peak [6,17]. Our secondary analyses also revealed that the vertical impact peak of rearfoot strikes occurred around 15% (mean ± SD: 14.78 ± 1.28%, calculated from 4000 steps) of the stance phase. The VALR and VILR were computed for the right and left legs and averaged over 200 consecutive steps for each leg. Then, we averaged the VALR and VILR calculated from the right and left leg. Further, because measure variability serves as an indicator of adaptation, we assessed whether imposing a running strike was more detrimental to usual rearfoot or forefoot strikers by computing the variance of the VALR and VILR over the 200 measures of VALR and VILR. As a secondary analysis, we verified whether runners adapted their running strike during the imposed trial. We divided the 200 steps analyzed into 10 blocks, each block representing 20 consecutive steps for both groups. Then, we averaged the VALR and VILR of each leg and calculated the variance. We reasoned that reducing the variance across the 10 blocks would suggest adaptation when running with an imposed foot strike. We performed this secondary analysis only on the imposed foot strike conditions that showed higher VALR or VILR variance than usual.

**Figure 2:**
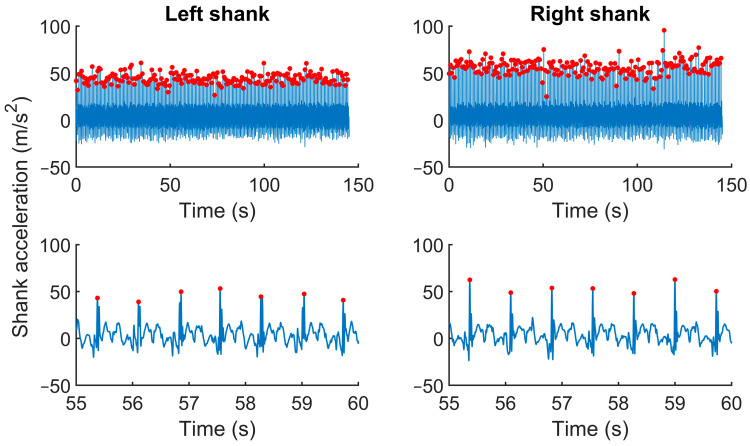
Typical shank acceleration time series for a rearfoot strike impact from one participant for left and right shank (upper panels). The red dots represent the shank acceleration peaks. The lower panels depict an insert of the left and right shank acceleration time series.

We applied the same procedure to analyze the peak shank acceleration to verify whether its amplitude and variance corresponded with the vertical loading rate data. From each shank acceleration time series, we identified the shank acceleration peak (i.e., along the long axis) over the 200 running cycles (Figure 2). Then, we averaged the peaks from the right and left legs. We computed the average shank acceleration peak’s amplitude and variance.

### 2.4. Statistical Analysis

We used nonparametric permutation testing to assess the influence of imposing a foot strike on the loading rate of both groups (i.e., usual rearfoot and forefoot strikers) [47,48]. We randomly shuffled (i.e., 10,000 permutations) condition labels (i.e., usual and imposed). At each permutation, for each dependent variable (i.e., VALR and VILR amplitude or variance and shank acceleration peak amplitude or variance), we computed *t*-test values to build the empirical distribution of the differences expected by chance. We compared this empirical distribution to the observed differences between the usual and imposed conditions for each group and dependent variable. Finally, to compute a *p*-value for each dependent variable, we calculated the z-scores: the difference between the usual and imposed foot strike conditions and the mean of the empirical distribution divided by the standard deviation of the empirical distribution. To evaluate if participants adapted their VALR or VILR during the imposed foot strike condition, we also performed nonparametric permutation testing, but this time, between the variability of the 1st and the 10th blocks for each group. We used the same procedure explained above but considered the 1st and 10th blocks as the independent variables. We performed the statistical analyses using custom-made MATLAB scripts (version 2020b, The Mathworks Inc., Natick, MA, USA). We set the statistical significance to *p* < 0.05. Because we used nonparametric permutation testing, the data depicted in the figure are represented by medians and the 25th and 75 percentiles.

## 3. Results

**Figure 3:**
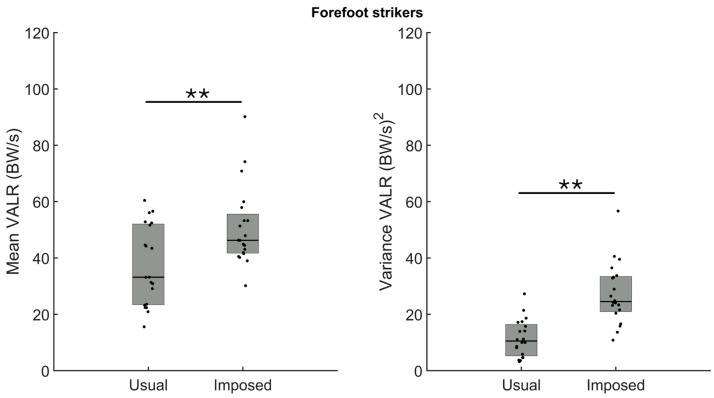
Group medians (n = 20) for the amplitude (**left panel**) and the variance (**right panel**) of the vertical average loading rate (VALR) when participants ran with their usual forefoot strike (usual) or with an imposed rearfoot strike pattern (imposed). Dots represent mean results for each participant; the horizontal lines depict the medians, and the boxes represent the 25th and 75th percentiles. ** Indicates statistical significance between both conditions (*p* < 0.01).

For the participants running with a usual forefoot strike pattern (n = 20), the comparison of the amplitude of the VALR between the usual and imposed patterns revealed a larger loading rate amplitude during the imposed foot strike (Figure 3, left panel, *p* = 0.004). The analysis also confirmed a larger VALR variance when running with the imposed foot strike than the usual foot strike (Figure 3, right panel, *p* < 0.001).

**Figure 4:**
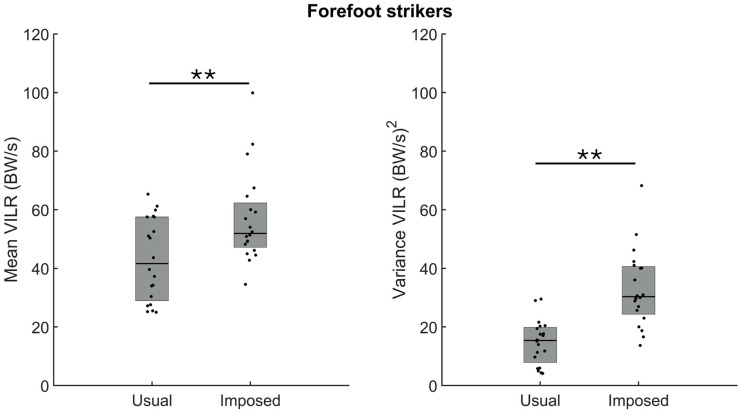
Group medians (n = 20) for the amplitude (**left panel**) and the variance (**right panel**) of the vertical instantaneous loading rate (VILR) when participants ran with their usual forefoot strike (usual) or with an imposed rearfoot strike pattern (imposed). Dots represent mean results for each participant; the horizontal lines depict the medians, and the boxes represent the 25th and 75th percentiles. ** Indicates statistical significance between both conditions (*p* < 0.01).

The analysis of the amplitude and the variance of the VILR between the usual forefoot strike and imposed rearfoot strike patterns confirmed a larger amplitude (Figure 4, left panel, *p* = 0.004) and variance (Figure 4, right panel, *p* < 0.001) of the VILR when running with an imposed rearfoot strike pattern.

**Figure 5:**
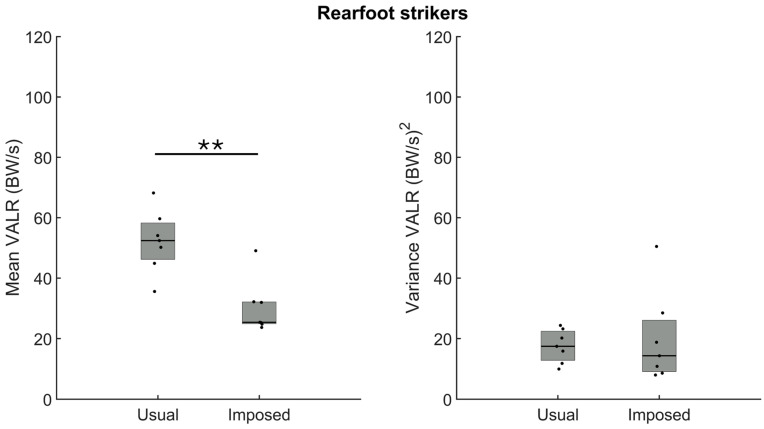
Group medians (n = 7) for the amplitude (**left panel**) and the variance (**right panel**) of the vertical average loading rate (VALR) when participants ran with their usual rearfoot strike (usual) or with an imposed forefoot strike pattern (imposed). Dots represent mean results for each participant; the horizontal lines depict the medians, and the boxes represent the 25th and 75th percentiles. ** Indicates a statistically significant difference between both conditions (*p* < 0.01).

For the rearfoot strikers, the analysis revealed a larger VALR amplitude during running with a usual rearfoot pattern (n = 7) than during the imposed forefoot strike (Figure 5, left panel, *p* = 0.002). For this group, however, there was no difference in the variance of the VALR when running with the usual or the imposed foot strike pattern (Figure 5, right panel, *p* > 0.05).

**Figure 6:**
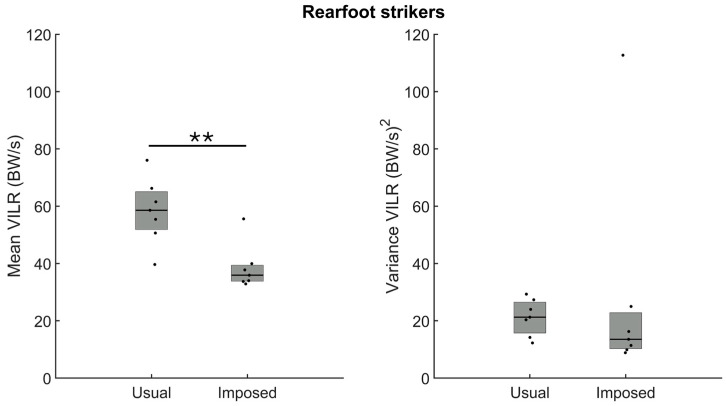
Group medians (n = 7) for the amplitude (**left panel**) and the variance (**right panel**) of the vertical average loading rate (VILR) when participants ran with their usual rearfoot strike (usual) or with an imposed forefoot strike (imposed). Dots represent mean results for each participant; the horizontal lines depict the medians, and the boxes represent the 25th and 75th percentiles. ** Indicates a statistically significant difference between both conditions (*p* < 0.01).

The analysis of the amplitude of the VILR confirmed larger loading when running with the usual rearfoot than the imposed forefoot strike pattern (Figure 6, left panel, *p* = 0.003). As for the VALR, the variance of the VILR was similar; we observed no difference in the variance between the usual and imposed foot strike patterns (Figure 6, right panel, *p* > 0.05).

**Figure 7:**
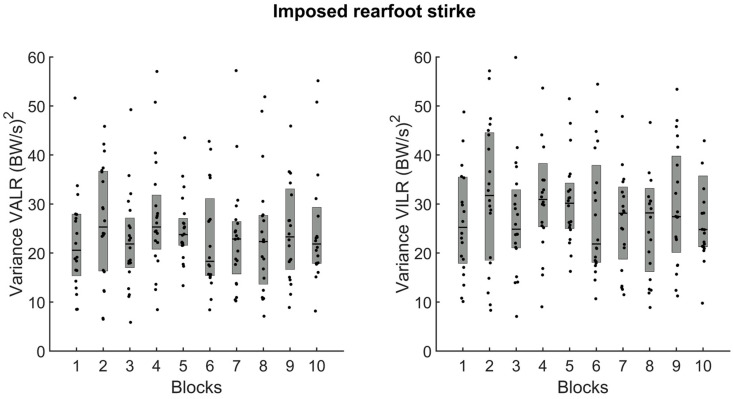
Variance for the VALR (**left panel**) and VILR (**right panel**) when participants ran with an imposed rearfoot strike pattern (n = 20). In each panel, the dots represent the mean results for each participant, horizontal lines represent the medians, and the boxes represent the 25th and 75th percentiles.

We performed a secondary analysis on the imposed rearfoot strike conditions to determine whether loading rate variance decreased during trials. The analysis (Figure 7) confirmed no change in the variance for the VALR (left panel) or the VILR (right panel) between the first and tenth blocks when imposing a rearfoot strike (Figure 7, *p* > 0.05). Visual inspection of Figure 7 confirms the outcome of the statistical analysis; there were no drastic changes in the variance of VALR or VILR across blocks.

**Figure 8:**
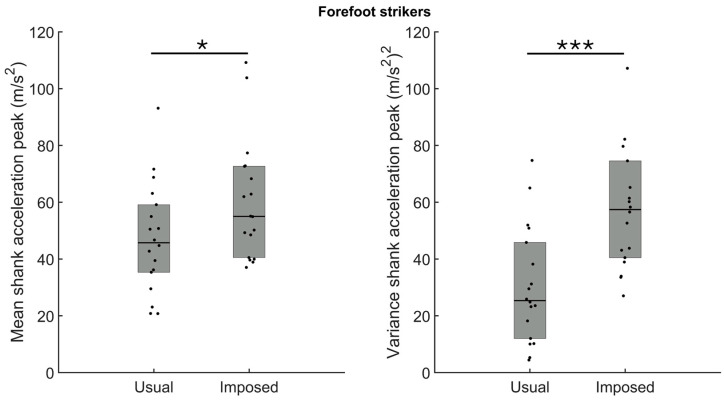
Group medians (n = 18) for the mean shank acceleration peak (**left panel**) and the variance shank acceleration peak (**right panel**) when participants ran with their usual forefoot strike (usual) or with an imposed rearfoot strike (imposed). Dots represent mean results for each participant; the horizontal lines depict the medians, and the boxes represent the 25th and 75th percentiles. * Indicates a statistically significant difference between both conditions (*p* < 0.05); *** indicates a statistically significant difference between both conditions (*p* < 0.001).

The amplitude and variance of the shank acceleration peak for participants running with a usual forefoot strike were larger when they ran with an imposed rearfoot strike (Figure 8, *p* = 0.03 and *p* < 0.001, for the mean (left panel) and the variance (right panel), respectively).

**Figure 9:**
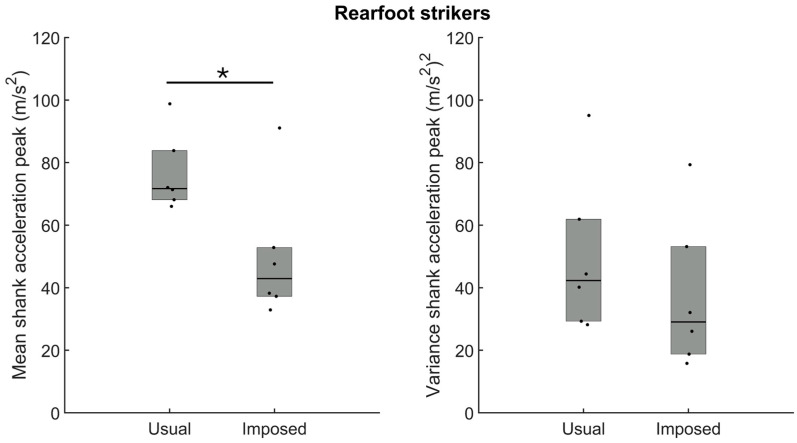
Group medians (n = 6) for the mean shank acceleration peak (**left panel**) and the variance shank acceleration peak (**right panel**) when participants ran with their usual rearfoot strike (usual) or with an imposed forefoot strike (imposed). Dots represent mean results for each participant; the horizontal lines depict the medians, and the boxes represent the 25th and 75th percentiles. * Indicates a statistically significant difference between both conditions (*p* < 0.05).

For the rearfoot strikers, the amplitude of the shank acceleration peak was reduced significantly when participants ran using a forefoot strike compared to the usual rearfoot strike (Figure 9—left panel, *p* = 0.02). However, as for VALR and VILR, the shank acceleration peak variance was unaffected when the participants ran with an imposed forefoot strike (Figure 9—right panel, *p* > 0.05).

## 4. Discussion

This study compared runners’ loading rate and shank acceleration peak amplitude and variability while running with either usual or imposed foot strikes. Because running pattern retraining is often employed to reduce loading impact, we propose that altering the foot strike pattern likely leads to an immediate increase in loading rate or shank acceleration peak variabilities. The increase in variability may harm the lower-limb structure, as low variability indicates the stress is concentrated on the same structure, and high variability suggests the neuromuscular system may not be adequately prepared to handle the impact. Further, we hypothesized that the loading rate or shank acceleration peak amplitudes would be higher when running with a rearfoot strike than a forefoot strike. Partially consistent with our hypothesis, the results confirmed a more significant loading rate or shank acceleration peak variabilities when running with an imposed rearfoot than the usual forefoot strike. However, loading rate or shank acceleration peak variabilities did not increase when running with an imposed forefoot strike compared to the usual rearfoot strike. Also, the amplitude of the loading rates and shank acceleration peak was higher when running with a rearfoot strike pattern, regardless of the foot strike conditions (i.e., usual or imposed). The current results indicate that adopting a forefoot pattern does not change the loading rate or shank acceleration peak variabilities for long-term rearfoot strikers while significantly reducing their amplitudes. Conversely, imposing a rearfoot pattern on long-term forefoot strikers increased the loading rate and the shank acceleration peak amplitudes and variabilities. Overall, gait retraining employing a forefoot strike reduces the loading rate and shank acceleration peak amplitudes, as already known, and should not increase the loading rate or shank acceleration peak variabilities.

The participants in our study mostly ran using a non-rearfoot (i.e., forefoot or midfoot) strike as their usual foot strike. This striking pattern contrasts with previous studies, where the rearfoot strike was the most used foot strike among recreational or sub-elite runners during long-distance races [49,50]. Many of the participants recruited (n = 12) were Canadian University athletes in athletics; therefore, they commonly use a forefoot strike for a more efficient running economy and better performance to optimize their running velocities [51].

In agreement with our results, previous studies reported larger loading rate amplitude when running with rearfoot than forefoot strikes [17,20,52]. The impact peak produced early in the stance phase during a rearfoot strike explains the differences in loading rate and shank acceleration peak amplitude [6,18,20]. Hence, loading rate and shank acceleration peak amplitudes depend more on the foot strike pattern than on the imposition of a usual or unusual foot strike. In our study, the vertical loading rates were lower (~20 to 30 BW/s and 15 BW/s for usual rearfoot and forefoot, respectively) than in previous studies [6,21]. In these studies, however, the authors calculated the vertical loading rates (i.e., VALR or VILR) using only a single step or non-sequential steps from several trials during overground running. In the current study, we averaged the vertical loading rate over 200 steps during treadmill running, which likely produced a lower vertical loading rate than overground running [53].

The vertical loading rate (i.e., VALR and VILR) and shank acceleration peak variabilities were larger during the imposed rearfoot strike running. Moreover, we observed no change in the vertical loading rate variability between the 1st and 10th blocks during the imposed rearfoot strike trial; hence, no adaptation occurred during this condition. This latest observation partially contrasts with a previous study reporting no differences in knee or ankle joint stiffness in runners using an imposed foot strike compared to runners using their usual foot strike pattern independently of the foot strike pattern (i.e., forefoot or rearfoot) [41]. However, this study calculated lower-limb joint stiffness using 10 foot strikes, and changes in joint stiffness variance were not the aim of this study. Another study measured the variability of vertical loading rate (i.e., standard deviation) across 6 foot strikes in novice barefoot runners [33]. Nonetheless, it did not examine the variability of vertical loading rate following alterations in foot strike pattern. The results revealed that barefoot running generated more considerable loading rate variability than running with shoes. The authors reported that novice barefoot runners may not have accustomed themselves to absorbing the GRFs without shoe cushioning. Similarly, our study asked participants to switch to a newly imposed foot strike pattern. Changes in foot strike patterns require different adaptations, as forefoot running increases ankle joint loading [42,54,55], while rearfoot running increases knee joint loading [41,42,43,56]. Studies showed that runners exaggerate their foot strike pattern when switching to an imposed foot strike [17,43]. However, these studies only analyzed a single step or steps during a 30 s trial; thus, it is challenging to conclude that participants continued exaggerating their foot strike pattern over time. In the current study, we analyzed the vertical loading rate and shank acceleration peak variabilities over 200 consecutive steps for each leg, which may explain differences in loading rate and shank acceleration peak variabilities in the imposed rearfoot strike condition. However, we observed no differences in the vertical loading rate or shank acceleration peak variabilities between the usual rearfoot strike and imposed forefoot strike, although the imposed forefoot strike showed a decreased loading rate and shank acceleration peak amplitudes. We do not have a clear explanation for the unchanged variance. The relationship between human joint damping and variance during running involves several factors (e.g., muscle activation and shoe type). Nonetheless, greater damping resists joint oscillations more effectively and leads to smoother joint movement [57]. Thus, running with an imposed forefoot strike can help fine-tune lower-limb joint damping and reduce joint movement variance (e.g., loading rate or shank acceleration peak). Moreover, typical vertical GRFs are similar for forefoot and midfoot strikes, where no impact peak occurred in the early stance phase [17,18]. Variations between these foot strikes from step to step result in small changes in vertical loading rates; this may not translate into higher loading rate variability in the imposed forefoot strike condition.

Our work had some limitations. First, participants ran on a treadmill at a constant speed without surface inclination, which may not completely reflect overground running. Second, participants had a limited familiarization period before acquiring data. Familiarization trials for each running condition lasted 2 min because of time restrictions. A more extended familiarization period (i.e., several training sessions) could help participants adapt to a short-term imposed rearfoot strike pattern and reduce the vertical loading rate variability. A study reported that habitual shoe runners familiarized themselves with barefoot running within 11 to 20 min [58]. Further, researchers noted that there is an optimal shoe type for running with rearfoot and forefoot strikes [19]. Hence, it is unclear if, besides imposing foot strikes, using the optimal shoe type would influence the current results. The loading rate or shank acceleration peak variabilities of forefoot strikers might not have increased if these runners had run with shoes built for the rearfoot strikers.

## 5. Conclusions

In conclusion, this study quantified the variability of vertical loading rate and shank acceleration peak across 200 consecutive steps while running using either usual or imposed foot strikes (i.e., forefoot or rearfoot). Switching to an unusual rearfoot strike increased the vertical loading rate and shank acceleration peak variabilities, whereas switching to an unusual forefoot strike did not increase the vertical loading rate or shank acceleration peak variabilities. The research protocol of the current study did not enable us to identify the optimal loading rate or shank acceleration peak variabilities that could help reduce running-related injuries. Thus, future studies must assess whether variability in loading rate or shank acceleration peak is associated with running-related injuries and identify if an optimal amount of loading rate or shank acceleration peak variabilities exists to minimize these injuries, as previously proposed for movement or sports tasks [30,31]. Moreover, future studies should assess whether efficient tuning of lower-limb joint damping explains the reduced variance during forefoot strikes.

## Figures and Tables

**Table 1:** Demographics characteristics of participants (mean ± SD).

	Male (n = 16)	Female (n = 11)
Age (y)	22.8 ± 2.9	22.1 ± 3.1
Height (m)	1.78 ± 0.06	1.62 ± 0.1
Body mass (kg)	68.1 ± 9.3	58.0 ± 4.2
Distance (km/week)	57.5 ± 36.7	37.3 ± 23.5
Speed (m/s)	2.78 ± 0.31	2.45 ± 0.35
Usual foot strike	13 FFS, 3 RFS	7 FFS, 4 RFS

Abbreviations: FFS = usual forefoot or midfoot strikers; RFS = usual rearfoot strikers.

## Data Availability

The data that support the findings of this study are available on reasonable request from the corresponding author. The data are not publicly available due to privacy or ethical restrictions.

## Supplementary Materials

The following supporting information can be downloaded at: https://www.mdpi.com/article/10.3390/s24248163/s1. Figure S1: Group-average power spectrum density of the ground reaction force (GRF) data for usual (left panel) and imposed (right panel) conditions.
