# Supplementary material for: Influence of Sudden Changes in Foot Strikes on Loading Rate Variability in Runners
Source: Sensors (Basel). 2024 Dec 21;24(24):8163. doi: 10.3390/s24248163 (PMC11679124; doi:10.3390/s24248163)
Supplement: Supplementary file 1 [file sensors-24-08163-s001.zip › sensors-3339482-supplementary.pdf]

To determine the cut-off frequency of the low-pass filter, we calculated the ground reaction force's power spectrum density (Fig. S1). The figure depicts the group-average power spectrum density for the usual and imposed foot strike conditions. Peak power occurred below 15 Hz for both foot strike conditions. Hence, we chose a cut-off frequency of 20 Hz.

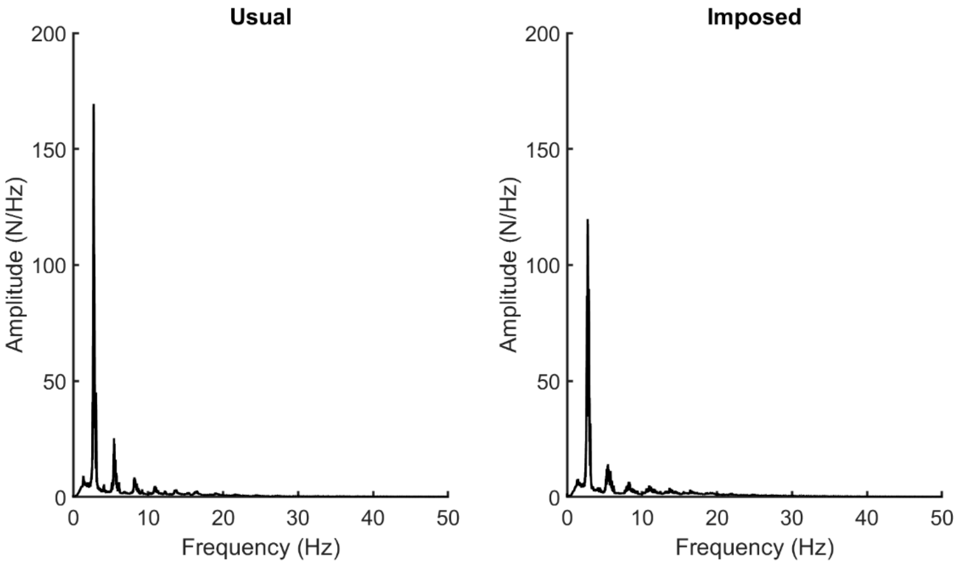

Figure S1: Group-average power spectrum density of the ground reaction forces (GRF) data for usual (left panel) and imposed (right panel) conditions.
